# Assessing spermatozoal small ribonucleic acids and their relationship to blastocyst development in idiopathic infertile males

**DOI:** 10.1038/s41598-022-24568-w

**Published:** 2022-11-21

**Authors:** Matthew Hamilton, Stewart Russell, Karen Menezes, Sergey I. Moskovtsev, Clifford Librach

**Affiliations:** 1 CReATe Fertility Centre, Toronto, ON Canada; 2 Department of Laboratory Medicine and Pathobiology, University of Toronto, Toronto, ON Canada; 3 Department of Obstetrics and Gynecology, University of Toronto, Toronto, ON Canada; 4 Department of Physiology and Institute of Medical Sciences, University of Toronto, Toronto, ON Canada; 5 Sunnybrook Research Institute, Toronto, ON Canada

**Keywords:** RNA, Diagnostic markers, Molecular medicine, Gonads, Male factor infertility, Embryogenesis

## Abstract

Clinical testing strategies for diagnosing male factor infertility are limited. A deeper analysis of spermatozoa-derived factors could potentially diagnose some cases of ‘unexplained infertility’. Spermatozoa carry a rich and dynamic profile of small RNAs, which have demonstrated potential developmental importance and association with fertility status. We used next-generation sequencing to correlate sperm small RNA profiles of normozoospermic males (n = 54) with differing blastocyst development rates, when using young donor oocytes. While ribosomal RNAs accounted for the highest number of sequencing reads, transfer RNA fragments of tRNA^Gly/GCC^ and tRNA^Val-CAC^ were the most abundant sequences across all sperm samples. A total of 324 small RNAs were differentially expressed between samples with high (n = 18) and low (n = 14) blastocyst rates (*p*-adj < 0.05). Ninety three miRNAs were differentially expressed between these groups (*p*-adj < 0.05). Differentially expressed transfer RNA fragments included: 5'-tRF-Asp-GTC; 5'-tRF-Phe-GAA; and 3'-tRF-Ser-GCA. Differentially expressed miRNAs included: let-7f-2-5p; miR-4755-3p; and miR-92a-3p. This study provides the foundation on which to validate a clinical panel of fertility-related sperm small RNAs, as well as to pursue potential mechanisms through which they alter blastocyst development.

## Introduction

Infertility has a considerable global impact, affecting roughly 15% of individuals and couples of reproductive age, and can have profound financial and psychosocial consequences for individuals undergoing fertility treatment^1,2^. Female partners experience the largest burden during clinical management of infertility, often undergoing laboratory testing, ultrasonography, hysterosalpingography, and surgical procedures^3^. However, male etiological factors are estimated to contribute to 50% of infertility, and are the sole cause in 20–30% of cases^4^. Therefore, effective diagnosis and management of male factor infertility is critical.

Many cases of male infertility are attributed to low sperm counts (oligozoospermia), low motility (asthenozoospermia), morphological deformities (teratozoospermia), male anatomic abnormalities or hormonal abnormalities^5^. These types of etiologies are routinely discerned through a reproductive history, a physical examination, blood testing and semen analyses^6,7^. However, the underlying cause of male subfertility is unidentified in 30–50% of cases, suggesting there may be uncharacterized spermatozoal defects, which are not adequately assessed by the current diagnostic work-up^4,8^. Thus, a more comprehensive analysis of sperm-borne genomic and extragenomic contents could be valuable.

It is increasingly clear that the developmental role of spermatozoa extends beyond simply transmitting the paternal haploid genome^9^. Despite translational inactivity, sperm carry an epigenetically-marked genome and a collection of ribonucleic acids (RNAs) and proteins^10^. Of particular interest, sperm carry a rich profile of small, non-coding RNAs, including microRNAs (miRNAs), PIWI-interacting RNAs (piRNAs), ribosomal RNAs (rRNAs) and transfer RNA (tRNA) fragments^11–13^. Numerous groups have suggested that the sperm small RNA profile is drastically altered as maturing sperm transition through the male reproductive axis^14–18^. Vesicles trafficked to maturing sperm in the epididymis, known as epididymosomes, have demonstrated the ability to deliver small RNAs to sperm, which may suggest selective packaging and delivery^14,19^. Furthermore, numerous studies have demonstrated plasticity of the sperm RNA profile in response to paternal toxin exposures, including exposure to dibutyl phthalate and bisphenol A; as well as changes in lifestyle, including short-term diet- and exercise-based interventions^20–25^. Such paternal exposures can modify the sperm RNA payload that is delivered to the oocyte at fertilization.

It is fairly well-established that sperm-borne RNAs are present in the embryo and persist throughout early development^26,27^. While the precise function of sperm small RNA elements in the developing embryo are unclear, they may influence oocyte activation, zygotic genome activation, regulate early embryonic gene expression, and affect offspring phenotypes^12,28^. In fact, animal studies suggest they are indispensable for normal developmental competence^27,29,30^. For instance, an sncRNA-deficiency, achieved via gene knockout, significantly reduced preimplantation development in mice, and this was rescued when sncRNA-deficient sperm was co-injected with either wild-type sperm total or small RNAs^29^.

Consequently, sperm-borne RNAs have garnered interest as clinical infertility markers. Numerous studies have correlated various sperm small RNAs with semen parameters, embryo quality, and fertility status in animals and humans^13^. In a clinical study, human sperm piRNAs (piR-31704 and piR-39888) were lower in males with oligoasthenozoospermia, compared to fertile controls, and higher in spermatozoa from couples with higher 2PN rates^31^. Moreover, increases in a number of human sperm miRNAs (miR-122 and miR-383) have also been noted in cases of moderate and severe oligoasthenoteratozoospermia^32^. In addition, specific tsRNAs (Gln-TTG) demonstrated association with sperm quality and 3PN outcomes^33^.

However, the diagnostic value of the sperm small RNA profile depends on its ability to distinguish idiopathic infertile males, who display poorer preimplantation embryonic developmental outcomes despite a normozoospermic presentation. Hua et al. observed that a number of tsRNAs, rsRNAs, and miRNAs are differentially expressed in human sperm in accordance with embryo quality, despite a normal semen-parameter assessment using traditional methodology^34^. Recently, a number of tRNA fragments (5' tRF-Glu-CTC, 5' tRF-Lys-CTT, and 5'tRF-Gly-GCC) were found to be increased in the seminal plasma from male partners of couples with multiple failed ART cycles^35^. Further validation is needed to develop a clinically useful and reproducible panel of differentially expressed small RNAs. If proven to be a reliable and efficient strategy, surveying sperm small RNAs, used in conjunction with the traditional male factor infertility diagnostic work-up, could reduce time to pregnancy and be leveraged to assess therapeutic strategies aimed at improving male reproductive health.

In the present study, sperm small RNA profiles of normozoospermic males with differing levels of preimplantation embryo development were analyzed via next-generation RNA-sequencing to determine whether males with idiopathic infertility have aberrantly expressed sperm small RNAs.

## Methods

### Study subjects

This study was approved by Veritas IRB (Quebec, CA; IRB protocol number 2021-2343-7435–1). Informed consent was obtained from all participants and methods were performed in accordance with the Human Assisted Reproduction Act (S.C. 2004, c. 2). Human semen samples were collected between April 2017 and August 2020 from a total of 54 male patients presenting to the CReATe Fertility Centre, Toronto, Canada, for fertility evaluation. Studied male subjects were either: (1) undergoing 50% of donor oocytes fertilized from each member of a same-sex male couple (50/50); or (2) a single sperm provider with donor oocytes. All patients included in the study were normozoospermic, according to standard semen analysis; males with a diagnosis of oligozoospermia, asthenozoospermia or teratozoospermia were excluded. Blastocyst rates were calculated by dividing the number of 2PN zygotes by the number of blastocysts obtained. For 50/50 couples, only couples with a minimum of 4 ova fertilized for each partner, and a minimum blast rate discrepancy of 35% were included. Samples with a blastocyst rate higher or lower than one standard deviation from the mean were designated as the high (Hi) and low (Lo) groups, respectively. The mean blastocyst rate of all included 50/50 couples and single sperm provider patients grouped together were used to group the single sperm provider patients. Single sperm provider patients within one standard deviation from the mean formed the average (Av) group. Patient characteristics and IVF data are summarized in Table 1.

**Table 1:** Patient Characteristics.

Subject	Age	Sperm status	Group	# Oocytes	# MII	# 2PN	# Blastocysts
Lo1	45	Frozen	Male-male	19	12	6	1
Hi2	45	Frozen	Male-male	18	12	12	10
Lo6	52	Frozen	Male-male	12	7	6	0
Hi7	42	Frozen	Male-male	12	0	4	3
Hi8	45	Fresh	Male-male	12	7	5	4
Lo9	49	Fresh	Male-male	13	6	5	2
Lo10	40	Frozen	Male-male	26	12	5	1
Av11	38	Frozen	Male-male	26	11	9	5
Hi12	43	Fresh	Male-male	6	5	5	4
Lo13	44	Fresh	Male-male	6	6	6	2
Av14	44	Fresh	Single male	9	5	3	2
Av16	44	Fresh	Single male	25	21	18	12
Lo17	64	Fresh	Single male	53	37	25	9
Lo18	38	Frozen	Single male	31	22	7	2
Lo19	54	Frozen	Single male	13	11	8	3
Lo20	34	Frozen	Single male	35	15	11	4
Lo23	37	Frozen	Single male	35	21	17	6
Lo24	47	Fresh	Single male	27	2	6	2
Av25	46	Fresh	Single male	30	15	11	7
Hi26	51	Fresh	Single male	22	13	12	10
Av27	43	Frozen	Single male	36		12	8
Hi28	59	Fresh	Single male	4	3	3	3
Lo29	50	Fresh	Single male	6	3	2	0
Av30	57	Fresh	Single male	22	18	10	6
Hi31	44	Fresh	Single male	3	3	3	3
Av32	35	Frozen	Single male	13	9	4	2
Hi33	44	Frozen	Single male	17	16	16	13
Hi34	42	Fresh	Single male	10	6	5	4
Lo35	38	Fresh	Single male	11	8	8	2
Av36	49	Fresh	Single male	21	17	10	5
Lo37	39	Frozen	Male-male	22	9	7	0
Hi38	31	Frozen	Male-male	20	12	10	7
Av39	34	Frozen	Male-male	12	8	7	4
Lo40	57	Frozen	Single male	16	10	7	1
Lo41	34	Frozen	Male-male	21	13	13	3
Hi42	38	Frozen	Single male	62	44	40	28
Av43	35	Frozen	Single male	19	16	12	8
Hi44	34	Frozen	Male-male	11	7	5	5
Hi45	44	Frozen	Single male	32	29	21	19
Av46	35	Frozen	Male-male	22	9	5	3
Hi47	36	Frozen	Male-male	21	20	13	10
Lo48	44	Fresh	Single male	22	17	11	1
Av49	40	Frozen	Male-male	23	21	18	12
Hi50	22	Frozen	Single male	25	15	12	9
Hi51	51	Frozen	Single male	38	33	28	23
Hi52	41	Frozen	Male-male	20	20	8	6
Lo53	36	Frozen	Male-male	20	20	9	3
Av54	37	Fresh	Single male	31	30	14	7
Av55	52	Fresh	Single male	21	16	14	7
Av56	36	Frozen	Single male	14	9	4	2
Av57	42	Fresh	Single male	17	15	15	6
Hi58	47	Fresh	Single male	26	23	17	14
Hi59	42	Frozen	Single male	9	8	7	6
Hi60	36	Frozen	Single male	14	12	9	8

### Sample preparation

Fresh ejaculate samples, collected for IVF, were allowed to liquefy at room temperature. A standard computer-aided semen analysis (CASA), using the HTM-CEROS Sperm Analyzer (Hamilton Thorne), was undertaken within 60 min of collection. Sperm concentration and motility were evaluated by CASA, while morphology was assessed manually adhering to WHO recommendations^36^.

Remaining sample volumes and corresponding de-identified data were collected by CReATe Biobank personnel. The CReATe Biobank is certified by the CTRNet Biobank Program. The collection and biobanking of biological materials for research was REB approved. Purified sperm was collected 24 h post-washing; samples were centrifuged at 420 g for 10 min for effective partitioning of seminal plasma from spermatozoa. The resulting spermatozoal pellet was resuspended with 0.5 mL of sperm wash medium and immediately frozen at − 80 °C for downstream assessment.

### RNA isolation

RNA isolation procedures were adapted from an established sperm RNA extraction protocol^37^. An input of 2 million sperm, estimated by a Countess™ 3 Automated Cell Counter (Invitrogen), was used to account for variance in sperm concentration. The RNeasy Kit (Qiagen) used in conjunction with the MiRNeasy Kit (Qiagen) allowed for the small RNA fraction to be obtained. Briefly, samples were lysed and homogenized and dilute ethanol was introduced to optimize binding conditions; QIAzol phenol/guanidine-based lysis and isolation was used. A silica-based membrane allowed for RNA binding and isolation, and impurities were washed away. Purified RNA was eluted in water. Starting RNA input was evaluated by Qubit™ microRNA Assay Kit (Invitrogen) prior to entering the library preparation workflow.

### Library preparation and sequencing

Small RNA libraries were prepared using the NEXTFLEX Small RNA-Seq Kit v3 (Bioo Scientific). An input of 2 ng of isolated small RNA was used for all samples. Small RNAs were ligated with adapters and sequential reverse-transcription and cDNA barcoding and amplification was performed. No rRNA depletion steps were included within this protocol. Pooled libraries were further size-selected using Pippin HT (Sage Science) to a range of 140–190 base pairs; this was performed to eliminate dimers which may have formed throughout library preparation and isolate small RNA species of interest. Final library traces were assessed using the Bioanalyzer 2100 (Agilent technologies). Resulting small RNA libraries had an average length of approximately 150 base pairs.

The NextSeq 550 Sequencing System (Illumina) was used for small RNA sequencing. Samples were divided into separate pools to prevent barcode overlap, as the number of samples exceeded the number of unique barcodes included in the NEXTFLEX Small RNA-Seq Kit. Pooled small RNA libraries were denatured, diluted according to the manufacturer’s protocols, and sequenced at a single-end read length of 75 bp.

### BioInformatics and data analysis

Data was analyzed as previously described by our group^38^. Briefly, samples were demultiplexed with Bcl2Fastq (Illumina) and adapters were removed with the FASTX-Toolkit. Multiqc (SciLifeLab) provided additional sequencing quality metrics and assessment of cleaned sequences. Low quality and short (< 18 nt) reads were removed from the analysis. Samples with below 200,000 clean reads were excluded from further analysis. Reads were then mapped to the latest available small RNA databases using Unitas, in the following order: tRNA database (22.03.2022), piRNA cluster database (22.03.2022), Ensembl (Release 88), EnsemblGenomes (Release 35), tRNA database (09.04.2019), SILVA rRNA databases (Release 132), miRBase (Release 22)^39–43^. Sequences generating a single read were removed and count matrices were assembled with awk scripts.

The top 100 most abundant sequences across all samples which failed to annotate to known small RNAs were further investigated. Some of the sequences were identified using BLAST® (NCBI) and through a survey of existing literature containing RNA sequencing small RNA annotation data. These re-annotated sequences are listed in supplementary Table S1. Our samples were then reannotated with Unitas and these re-annotated sequences were added to improve overall small RNA detection.

Data was imported into R (R Development Core Team 2013) for differential expression (DE) analysis with DESeq2^44^. Two sets of DE analyses were performed: (1) all annotated small RNAs, and (2) human miRNAs only. Counts were collapsed by unique annotations, excluding sequence variants (eg. isomiRs). The design formula included the categorical variables age, percentage of library annotated, sperm concentration and blastocyst development rate. Data was explored using principal component analyses (PC1, PC2 plots) and unsupervised hierarchical clustering analyses using Pheatmap (version 1.0.12) and the complete linkage method.

Functional gene ontology (GO)-enrichment analyses of miRNAs were completed through: (1) generating categorical lists of the most highly annotated (n = 10) miRNAs and the DE miRNAs (n = 10); 5 up-regulated and 5 down-regulated; (2) identifying a list of predicted gene targets via TargetscanHuman 8.0; and (3) conducting a pathway analysis search using the ShinyGO v0.75: Gene Ontology Enrichment Analysis tool^45,46^. The top 10 miRNA target genes with the highest cumulative weighted context +  + score (combining 14 features which determine a ranking of significant gene targets) were compiled into single gene lists for each category of interest^47^. Absolute values for cumulative weighted context +  + scores ranged from 0.033 to 2.67. The resulting lists of miRNA target genes were used to conduct pathway analysis and identify relevant functional genomics data. Pathways of these gene targets were further investigated using the Kyoto Encyclopedia of Genes and Genomes (KEGG)^48^. NCBI-GeneIDs were converted to KEGG identifiers and pathway searches were conducted using the KEGG Mapper Search tool (https://www.kegg.jp). The compiled list of gene targets of up-regulated, down-regulated and abundant miRNAs were further cross-referenced with human imprinted genes cataloged in Geneimprint (https://www.geneimprint.com/).

## Results and discussion

### Sequencing results and gene mapping

**Figure 1:**
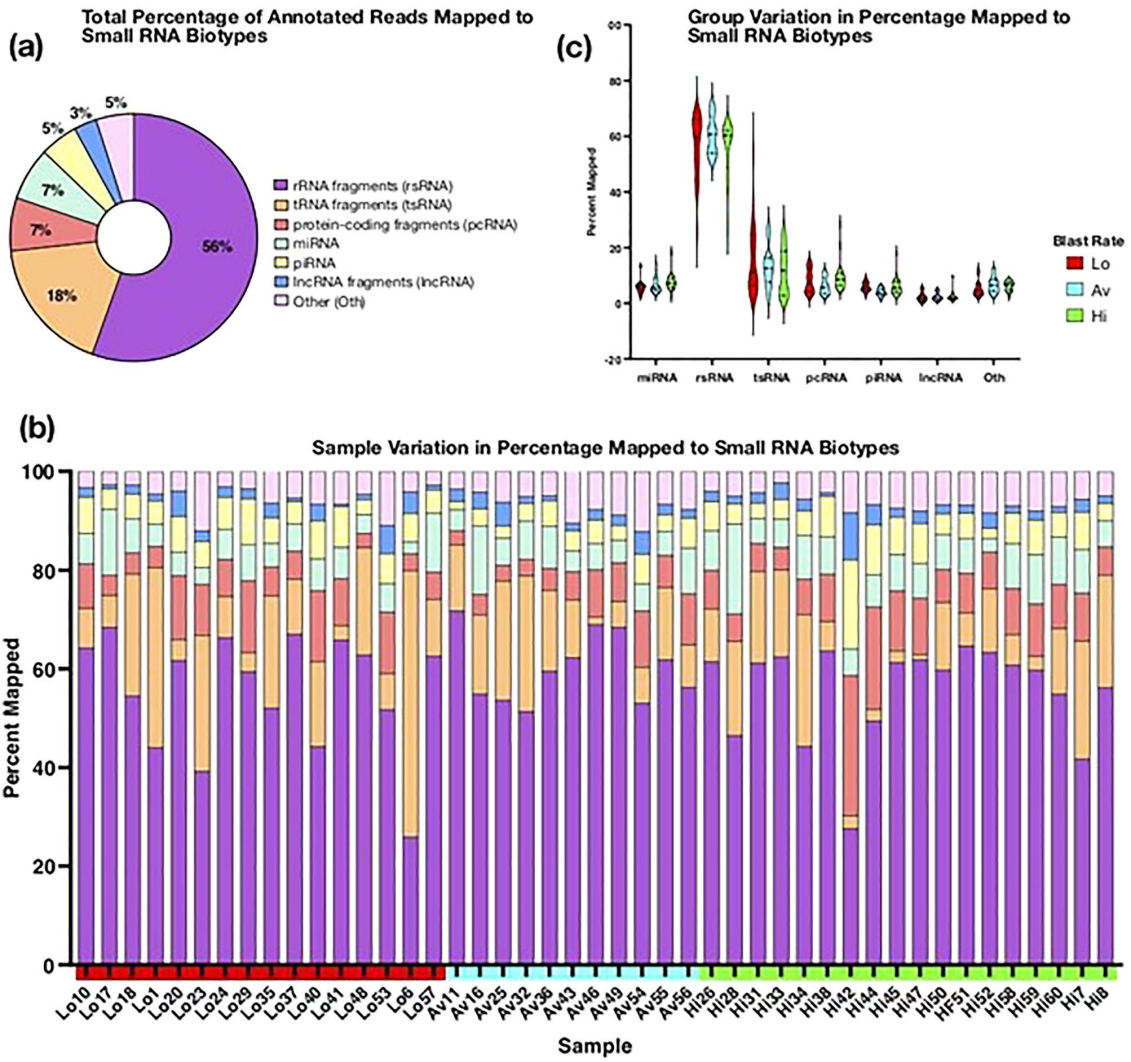
Categories of small RNAs identified. **(a)** Small RNA biotypes as a percentage of the total annotations. Small RNAs represented within ‘other small RNA,’ include miscellaneous coding and non-coding small RNAs, such as small nuclear RNA (snRNA) and mitochondrial genomic RNA. Both human miRNAs and sequences mapped to other miRNAs are included. **(b)** Small RNA biotype distribution across individual samples. The percentage of annotations mapped to rRNA fragments (mean = 57%; SD = 10%), tRNA fragments (mean = 14%; SD = 11%), miRNAs (mean = 7%; SD = 3%), piRNAs (mean = 6%; SD = 3%), protein-coding fragments (mean = 8%; SD = 5%), lncRNAs (mean = 2%; SD = 2%) and other small RNAs (mean = 6%; SD = 2%) are relatively uniform across samples, though tRNA and rRNA fragments demonstrate the highest variability. Hi, Av, and Lo refer to sample groupings (high, average and low, respectively), as indicated by blastocyst development rates. **(c)** Comparing Small RNA biotype distributions across the Hi, Lo, and Av groups. No significant differences in the mean biotype proportions for each blastocyst rate group were identified. A one-way ANOVA was used to compare groups.

Approximately 461 million raw reads were generated, with a mean of roughly 8.2 million reads per sample. In the samples analyzed, 55,338 unique annotated sequences were identified. The number of unique annotations did not differ among Hi, Av, and Lo groups (data not shown). Overall, the highest number of reads were mapped to rRNAs (55%), tsRNAs (18%)–including both genomic tsRNAs (14%) and mitochondrial tsRNAs (4%)–miRNAs (7%), protein-coding fragments (7%), and piRNAs (5%), and the remaining 7% of reads were attributed to other miscellaneous coding and non-coding small RNAs (Fig. 1a). However, the proportion of reads attributed to each of these small RNA biotypes varied among samples (Fig. 1b). For instance, tsRNA accounted for a range of 1.07% to 54.07% of sRNAs mapped for each sample. The small RNA biotype distributions were relatively uniform across Hi, Lo, and Av groups (Fig. 1c). The small RNA distribution identified across all samples is fairly consistent with previous sperm small RNA-seq studies in mice and humans, though some previously published library preparation protocols involved the depletion of rRNAs^49^. Our protocol, by contrast, captures all rRNA-derived sequences^50^.

Of the rRNAs identified in the present study, fragments of 28S and 5S rRNA were the most abundant. Elsewhere, 28S rRNA-derived small RNAs were markedly enriched in human mature sperm and associated with inflammation and the resulting subfertility in leukocytospermia patients^51^. Sperm are considered to be translationally inactive since they lack the proteins and organelles required to translate mRNAs stored in mature spermatozoa. While rRNA fragments present in mature sperm may simply be nonfunctional remnants from spermatogenesis, the abundance of rRNA fragments could also indicate targeted rRNA cleavage^52,53^. Cleaving rRNAs in sperm could function to either prevent mRNA translation post-spermiogenesis or direct protein synthesis in the zygote post-fertilization, though functional studies are lacking.

Indeed, protein-coding RNA fragments are also present (7% of total annotations). While protein-coding RNAs (and RNAs in general) in sperm are also believed to have no functional roles beyond spermatogenesis, their notable presence in mature sperm is intriguing^26^.

The piRNAs carried by spermatozoa are derived from the antisense strand of pseudogenes located within piRNA clusters^54^. The most abundant piRNA clusters identified in our dataset include: hsa-0088, hsa-0175, hsa-0084, and hsa-0142 (genomic locations indicated in extended names, supplemental Table S2). piRNAs are primarily thought to function in germline protection and integrity, though they may also contribute to sperm motility and post-transcriptional regulation of protein-coding genes^55^. However, only 5% of reads mapped to piRNAs. Other studies have also reported a minority of piRNAs in mature mammalian sperm, despite representing a considerable proportion in immature sperm in the testes and throughout spermatogenesis^15^. It has been proposed that dissociation of piRNA complexes that contribute to mRNA inactivation and remodeling in late spermiogenesis may render its associated piRNAs vulnerable to nuclease-mediated degradation^56^. The distributional shift of small RNAs may be partially explained by the considerable influx of tRNA fragments that reportedly occurs as maturing sperm passes through the epididymis^17^. Interestingly, a recent analysis of existing small RNA-seq databases obtained from the seminal plasma of normozoospermic males, reported that 88% of the sequences annotated as piRNAs are actually tRFs^35^.

Importantly, transfer RNA fragments from tRNA-Gly-GCC and tRNA-Val-CAC were the most abundant individual sequences present in our data, despite transfer RNA fragments, as a whole, only accounting for 18% of total reads. In fact, tRNA-Gly-GCC was present in all samples. These sequences have been reported elsewhere, in animal and in vitro studies, to impart developmental ability, and are suggested to be indispensable for successful preimplantation embryonic development^17,33,57^. Of the existing tsRNA types; referring to the region from which the tRNA molecule that the small RNA were derived, 5’ halves accounted for the largest number of annotations. 5' halves of specific tRNAs–tRNA^Gly/GCC^ and tRNA^Glu/CTC^–have been shown elsewhere to promote corresponding tRNA gene transcription in vertebrate (zebrafish) embryos; potentially contributing to protein synthesis during embryonic development via RNA elements delivered by sperm^58^. While the underlying mechanisms through which tRNA fragments reportedly influence zygotic activation and early embryonic development remain unclear, it has been suggested that they may direct transposon silencing^59^. Specifically, they may regulate embryonic expression of retroelements, including long interspersed nuclear elements and long terminal repeat retrotransposons, which may contribute to programming early embryonic development^57,60,61^. However, other mechanistic actions, including translation regulation and mRNA degradation, have also been proposed^59^. Functional studies of mouse embryonic stem cells have demonstrated that 5′-tRNA halves (derived from tRNA^Gly/GCC^, tRNA^Val/CAC^, tRNA^Gln/CTG^, tRNA^Glu/TTC^, and tRNA^Lys/TTT^) may regulate translation of the pluripotency‐promoting factor c‐Myc through RNA-binding protein (IGF2BP1) action; thereby regulating cell proliferation and metabolism^62,63^.

In total, 11,845 miRNA sequences were identified. With isoforms collapsed, these sequences were considerably reduced to 2011 unique human miRNAs. Top annotated miRNAs include: miR-12136-5p; miR-21-5p; miR-122-5p; miR-26a-5p; and miR-375-3p. A number of sources have identified miR-122 in sperm and seminal plasma, and have noted differences in its expression among infertile males^32,64^. Furthermore, miR-21-5p expression levels in sperm samples and spent culture media have been associated with embryo quality in couples undergoing IVF, with significantly lower miR-21-5p expression in samples yielding good quality embryos, compared to those with poorer quality embryos^65^. Over-expression of miR-21 in cumulus oocyte complexes has also been associated with increases in cleavage rate and blastocyst formation^66^. The let-7 miRNA family was also highly annotated. Mouse embryo studies have suggested that repression of the miR-let-7 family, via lin28a inhibition, and associated Wnt/β-catenin signaling, may promote embryo implantation, as well as the embryonic epithelial-mesenchymal transition^67^.

Long, non-coding RNA (lncRNA) and long intergenic, non-coding RNA (lincRNA) fragments accounted for 2% and less than 1% (0.73%) of total reads, respectively. However, a number of sequences mapping to lncAB370.3, lnc_000681, and lnc_000754 represented highly abundant sequences. While these specific lncRNAs have been reported elsewhere in RNA-seq supplementary datasets and have been identified in both spongiosa tissue and macrophages in humans (GenBank, National Library of Medicine), they lack functional characterization^68–70^.

The range of sequence lengths detected for each small RNA type, and overall (total small RNAs), are shown in supplementary Figure S1.

### Differential expression analysis

**Figure 2:**
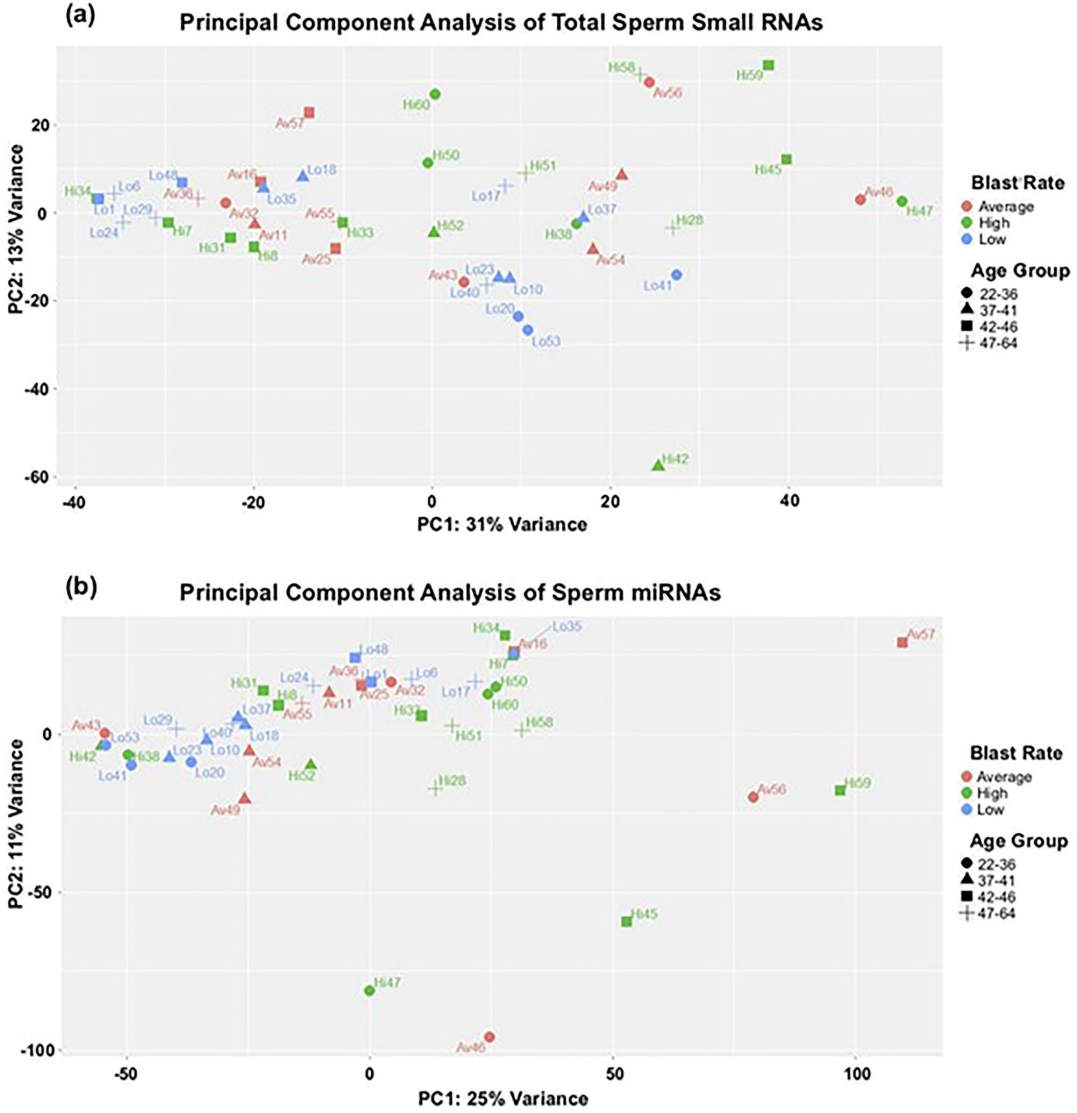
Principal Component Analysis. **(a)** Total small RNAs. The variation values of PC1 and PC2 are 31% and 13%, respectively. Plotted data points represent individual samples, which are labeled and coloured based on relative blastocyst rate. The age group of each sample is also depicted using shapes. **(b)** Human miRNA-only dataset. The values of PC1 and PC2 are 25% and 11%, respectively, and samples are represented in the same manner as (**a**).

Principal component analysis (PCA) was used to visualize the dimensionality-reduced small RNA differences between normalized samples to identify potential contributors to variation in the data (Fig. 2a). This process was also repeated for miRNAs, which are the most functionally well-characterized of the small RNA species (Fig. 2b). For total small RNAs, PCA demonstrated moderate clustering based on blastocyst rate, though the most effective clustering was observed in the low blastocyst rate group. Interestingly, clustering of samples by age was also noted. While fertility is known to decline with advancing maternal age, the relationship between paternal age and subfertility is less clear^71^. Specifically, the changes in the sperm RNA transcriptome that accompany paternal aging, and the related mechanisms, are not well characterized but may be related to neurodevelopmental disorders^72^. Though some studies have noted an overall decrease in genomic integrity with increasing paternal age, the apparent clustering in the intermediate age groups (ages 37–41; ages 42–46) noted in the present study differs from this trend^73^. While this could simply represent stochastic clustering, other studies have noted sperm small RNA variation amongst animals of particular ages^72,74^. For the miRNA-only analysis, considerable clustering of samples with low blastocyst rates was also noted, as well as the 37–41 and 47–64 age groups. Samples with high blastocyst rates also appear to cluster, similar to the low group.

**Figure 3:**
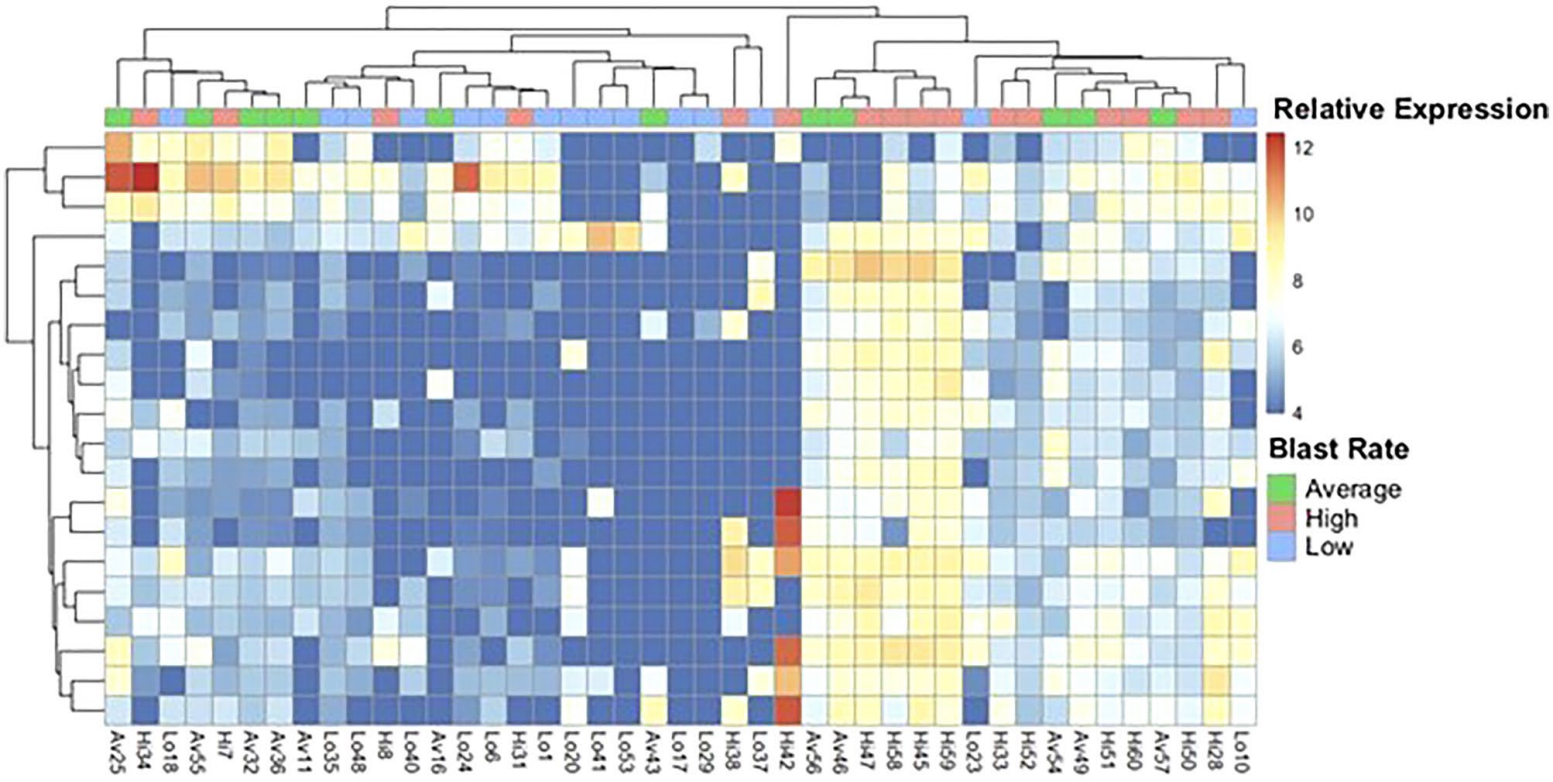
Heatmap of the top differentially expressed small RNAs. The top 20 differentially expressed small RNAs are shown for each sample, with unsupervised hierarchical clustering by sample and gene using Pheatmap (version 1.0.12) and the complete linkage method. Colors along the upper x-axis indicate the relative blastocyst rate of each sample. Relative expression is represented using the color scale shown on the right panel.

Importantly, 324 small RNAs were DE between the High (Hi, n = 18) and Low (Lo, n = 14) blastocyst rate groups (FDR adjusted *p* value of < 0.05). Moreover, 221 and 84 DE small RNAs were identified when comparing the Average (Av, n = 12) group with the Hi and Lo groups, respectively (*p*-adj < 0.05). Together, this data suggests that the Hi group has the most unique expression profile, particularly when compared with the Lo group. In fact, it was observed that most DE RNAs demonstrated markedly higher expression in the Hi group. This is a perhaps suggestive of a functional difference, in which the relative abundance of particular small RNA relative to the other groups confers developmental competence. Figure 3 shows a heatmap of the top 20 DE small RNAs. Considerable clustering of samples based on high and low blastocyst rate can be visualized, though the average blastocyst rate group is intermixed throughout. A complete list of small RNAs that were DE (*p*-adj < 0.05) among the Hi and Lo groups is included in the supplementary Table S3. tRNA fragments that were up-regulated in the Hi group, compared to the Lo group, include: 5'-tRF-Asp-GTC; 5'-tRF-Phe-GAA; 3'-tRF-Ser-GCA; 3'-tRF-Thr-TGT; and 3'-tRF-Leu-CCA. 5' tRNA-halves from tRNA^Leu/CAG^ and tRNA^Arg/TCG^ were both down-regulated in the Hi group. DE piRNA clusters were all up-regulated in the Hi group and include: hsa-0841; hsa-0861; and hsa-0877. Fragments of small subunit (SSU) rRNA and lincRNAs, including lincRNA-1694, lincRNA-2211, and lincRNA-957 were also DE. Over 100 protein coding RNA fragments were found to be DE, though many are from uncharacterized genes.

Many studies focus only on miRNAs and exclude other small RNA species, as they are the best studied and have the most well-defined regulatory functions. Therefore, we isolated the human miRNAs from our dataset, and performed a miRNA-specific analysis. We found 93 miRNAs DE between the Hi and Lo groups (*p*-adj < 0.05), 37 of which were present in at least 5 samples (supplementary Table S4). This number was further reduced to 7 when isolating only DE miRNAs present in a minimum of 10 samples. These specific miRNAs, with their average expression across blastocyst rate groups, are shown in Table 2.

**Table 2:** Top Differentially Expressed miRNAs.

Gene	Sample count*	Hi mean expression**	Lo mean expression**	Av mean expression**	Log2FoldChange***	*p*-adj.
let-7f-2-5p	10	95.27	2036.66	418.39	28.18	2.46E−16
miR-4755-3p	10	71.01	0.70	48.72	16.86	5.044E−06
miR-190b-5p	12	7.71	0.045	12.60	15.56	1.32E−06
miR-139-5p	14	0.11	33.96	7.682	13.53	2.077E−06
miR-19a-3p	11	21.06	0.35	6.30	9.41	0.046
miR-92a-3p	30	96.13	0	187.97	-5.39	0.026
miR-1343-5p	11	5.01	0.071	25.33	-12.79	0.00047

*Only differentially expressed small RNAs present in 10 or more samples are included.

**Expression values for the high, low and average blastocyst rate groups were normalized using a mean of ratios to allow for gene count comparisons between and within samples.

***Positive ‘log2foldchange’ values indicate up-regulation in the Hi group.

**Figure 4:**
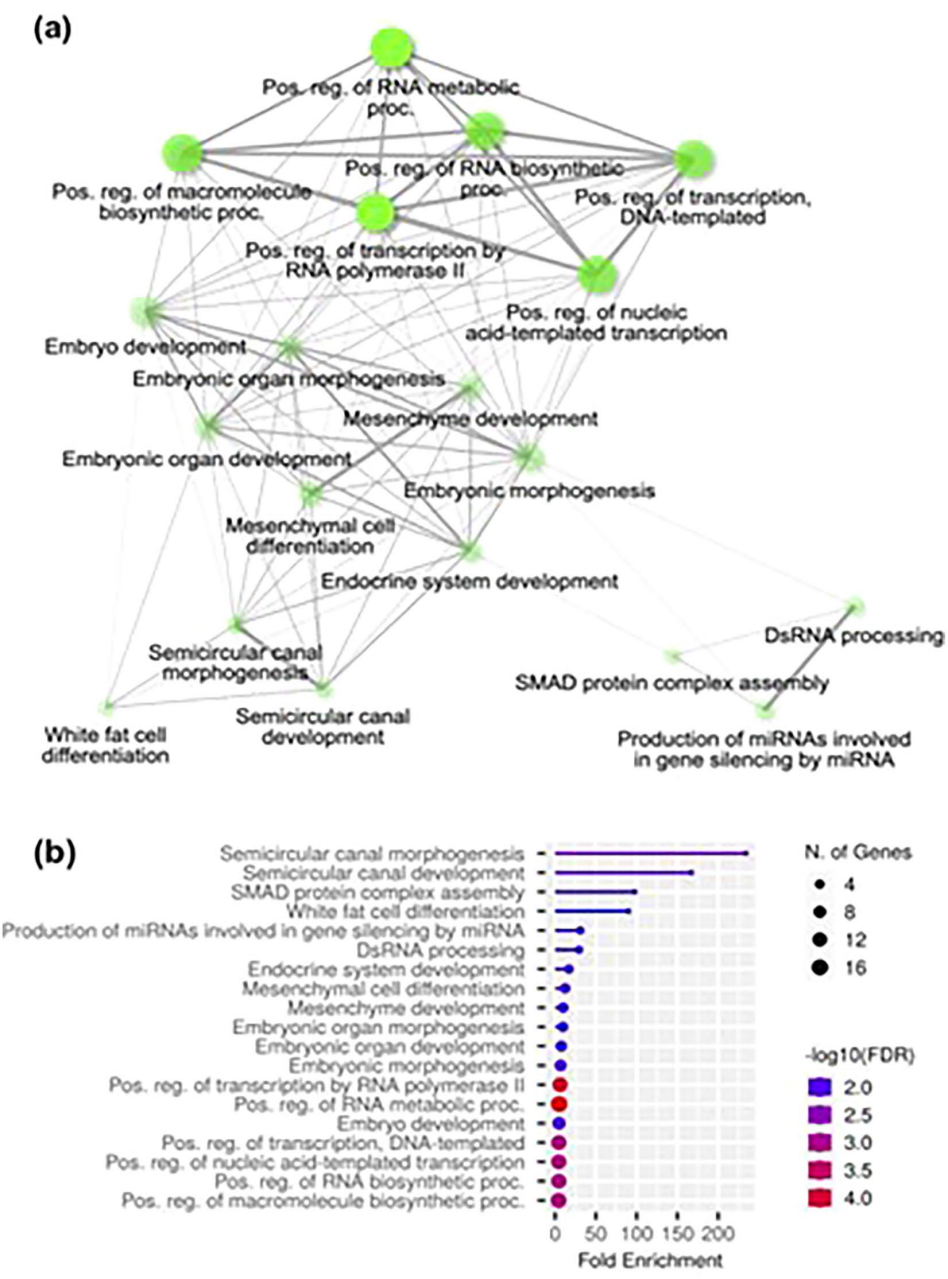
Gene Ontology (GO)-enrichment analysis of gene targets for up-regulated miRNAs. **(a)** Target genes of up-regulated miRNAs form a highly connected functional network. Apparent clustering of functions related to gene regulation, morphogenesis and development can be visualized. **(b)** High levels of enrichment are evident for the prominent pathways, with several target genes sharing common functional categories.

Of the top DE miRNAs, let-7f-2-5p, miR-4755-3p, miR-190b-5p, miR-139-5p, and miR-19a-3p were all up-regulated in the Hi group, while miR-92a-3p and miR-1343-5p were down-regulated. Several gene targets were identified for the most highly abundant miRNAs, up-regulated miRNAs, and down-regulated RNAs. The gene targets from each of these miRNA categories are listed in supplementary Table S5. GO-enrichment analysis of selected target genes (n = 40) influenced by the top up-regulated miRNAs suggests they may be important in processes such as embryogenesis and morphogenesis, as well as other regulatory signaling pathways and cellular and structural development (Fig. 4)^45^. The specific gene targets of up-regulated miRNAs contributing to enriched functional categories are listed in supplementary Table S6. Go-enrichment analysis of gene targets (n = 60) of highly abundant miRNAs showed a similar involvement in development and morphogenesis, however, no substantial enrichment was found through analyzing the gene target list (n = 50) for the most down-regulated miRNAs. KEGG pathway analysis supports the involvement of these gene targets of abundant and DE miRNAs in metabolic and cellular signaling pathways, as well as pathways of various disease states (supplementary Table S7). From the list of over 100 gene targets of up-regulated, down-regulated and abundant miRNAs, no known imprinted genes were identified.

## Conclusion

Here, we identified differences in sperm small RNA profiles of normozoospermic males who had embryos with varying pre-implantation developmental success post-ICSI. Specifically, a number of small RNA sequences were markedly up- or downregulated in idiopathic infertile males, when comparing differences in their ability to produce blastocysts. Alterations of these various small RNAs (including miRNAs, piRNAs, tRNA, rRNA, protein-coding RNA and lncRNA fragments) could potentially explain variances in embryo development among males with seemingly normal sperm.

The results are limited by a moderate sequencing depth. Furthermore, fertility groups were determined by blastocyst rate, taking into account the number of blastocysts resulting from mature (MII) donor oocytes. Confounding variables in this group may include: technical skill of the embryologist; embryo culturing conditions, which have been shown to influence blastocyst development; and maternal factors, despite the use of viable oocytes retrieved from young, healthy donors. Furthermore, the data was parsed by a number of covariates beyond blastocyst rate, including, sperm concentration, percent mapped to the genome, age, and post-extraction RNA concentration. While no substantial PCA clustering was observed in most cases, moderate clustering based on paternal age is an interesting finding, suggesting that the sperm RNA profile is distinct among certain age groups. Our study was limited by a higher-than-average patient age (42.7 years) in the demographic of interest (males utilizing donor oocytes) when compared to infertile patients as a whole. Further work to investigate the relationship between paternal age and the sperm sncRNA transcriptome is in progress. The observed clustering based on blastocyst rate supports our contention that the identified differences in the small RNA profile are indeed related to fertility outcomes of interest.

Given some of the limitations of our findings, future studies are required to validate the differentially expressed small RNAs we identified, and evaluate the clinical utility of our small RNA panel for diagnosing male infertility. Moreover, in future investigations we will assess how changes in sperm small RNAs delivered to oocytes impact early embryo development, and the underlying mechanisms for small RNA-derived effects. Other sperm related factors, including large coding and non-coding RNAs, proteins and epigenetic signatures also warrant further investigation. This could allow us to gain a holistic perspective of how sperm-derived factors intersect to influence fertilization and development.

With additional validation, a clinically-useful panel of differentially expressed sperm small RNAs could potentially be used to better assess male factor infertility and predict IVF success. Such diagnostic improvements could greatly reduce time to pregnancy for individuals and couples attempting to conceive, while mitigating the considerable psychosocial impacts of infertility and fertility treatment. In addition to their diagnostic utility, sperm-borne small RNAs which influence development to blastocyst may be helpful proxies for improving sperm quality through lifestyle or pharmacologic interventions.

## Supplementary Information

Supplementary Information 1.

Supplementary Information 2.

Supplementary Information 3.

Supplementary Information 4.

Supplementary Information 5.

Supplementary Information 6.

Supplementary Information 7.

Supplementary Information 8.

## Data Availability

The sequencing read data may be accessed through NCBI under BioProject PRJNA862094 and can be accessed at https://dataview.ncbi.nlm.nih.gov/object/PRJNA862094?reviewer=18fgtvcv8dntjg8o3t630um45j.
