# Supplementary material for: Assessing spermatozoal small ribonucleic acids and their relationship to blastocyst development in idiopathic infertile males
Source: Sci Rep. 2022 Nov 21;12:20010. doi: 10.1038/s41598-022-24568-w (PMC9678953; doi:10.1038/s41598-022-24568-w)
Supplement: Supplementary file 1 — Supplementary Information 1. [file 41598_2022_24568_MOESM1_ESM.docx]

**
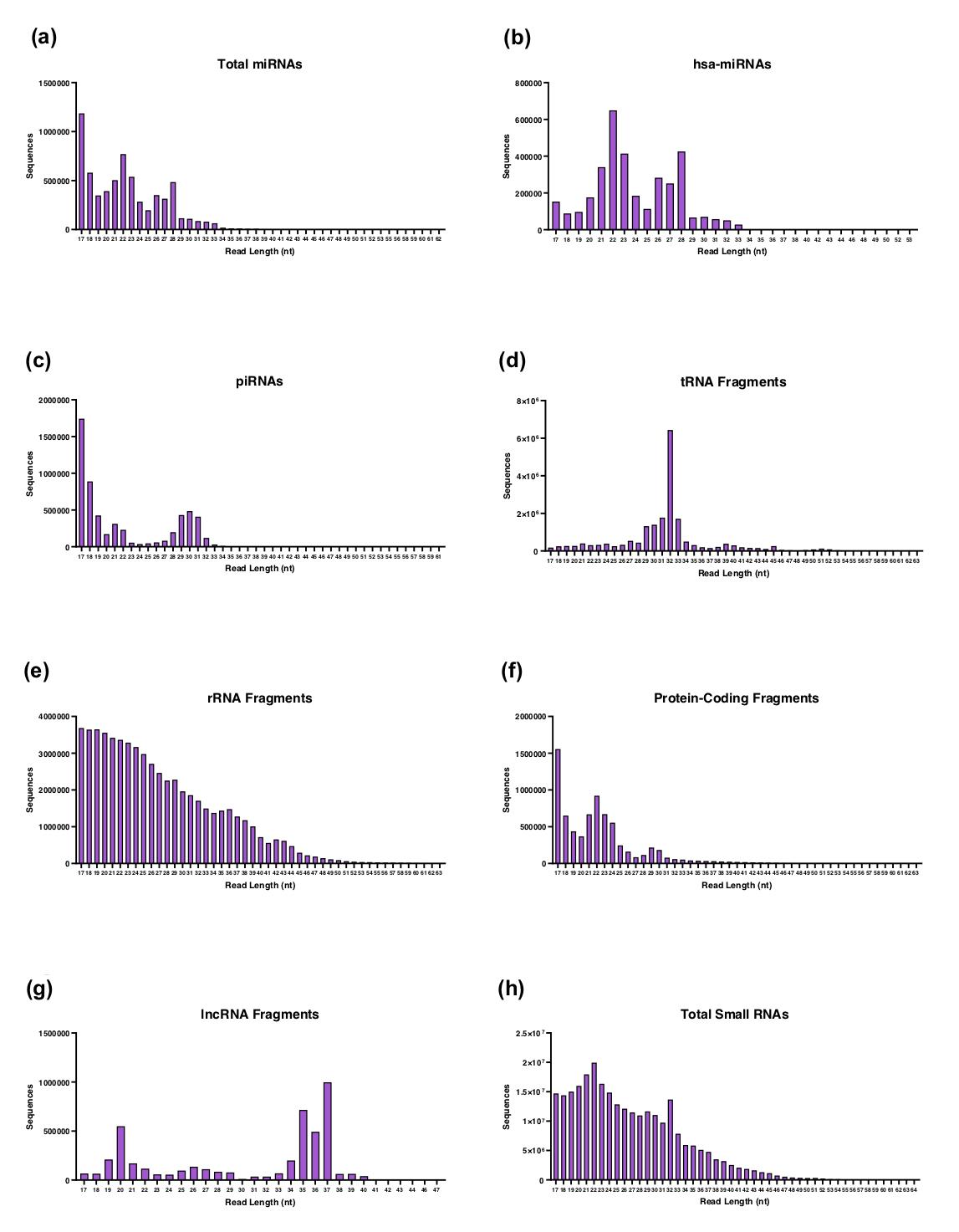
**

**Supplementary Figure S1. Read length distributions for small RNAs. (A-G)** Read lengths for different small RNA types of interest**.** While a large number of 17nt sequences may be noted in the total miRNA plot **(A)**, this outlier is greatly reduced when isolating solely miRNAs from *homo sapiens* **(B)**. **(H)** Read lengths for total small RNAs. Two prominent peaks may be noted at roughly 22nt and 32nt, representing miRNAs and tRNA fragments, respectively. An average sequence of roughly 26 nucleotides (nt), ranging from 17-64nt, was observed across all small RNAs.
